# The challenges in schizophrenia treatment in real-life: The uncomfortable truth

**DOI:** 10.1192/j.eurpsy.2021.125

**Published:** 2021-08-13

**Authors:** E. Rancans

**Affiliations:** Department Of Psychiatry And Narcology, Riga Stradins University, Riga, Latvia

**Keywords:** Treatment, Antipsychotics, schizophrénia

## Abstract

Certain percentage of the first-episode schizophrenia patients presents with negative symptoms, which persists over the year and influence treatment outcomes (Galderisi et al. 2013). Treatment of negative symptoms has been a significant continuous clinical challenge. Majority of recently published guidelines recommend antipsychotic monotherapy as the standard of care, recommending antipsychotic combination therapy only after a failed trial with clozapine (George A. Keepers et al. 2020; Faden et al. 2020). However, real-life forces clinicians to look for possible combinations of medications early on, especially to tackle negative symptoms. The systematic review of global prescribing practices covering four decades found the pooled median rate of antipsychotic combination therapy approximately 20% (Gallego et al. 2012). One of the largest retrospective studies every conducted (n = 62,250) assessed rehospitalisation rates and the long-term use of antipsychotic polypharmacy in schizophrenia. Antipsychotic combination treatment was associated with an approximate 10% lower relative risk of psychiatric rehospitalisation compared with antipsychotic monotherapy (Tiihonen et al. 2019). Real-world effectiveness study of antipsychotic monotherapy vs. polypharmacy in schizophrenia from Eastern Europe is also supporting this approach (Katona, Czobor, and Bitter 2014). At the same time antipsychotic combination therapy can increase the total antipsychotic dose burden, frequency of adverse effects, potential drug-drug interactions and incur additional costs. In our recent naturalistic study in schizophrenia outpatients (n=120) with insufficient effectiveness of previous antipsychotics therapy on negative symptoms, we were able successfully switch therapy form several different antipsychotic combinations to monotherapy and gain clinical benefits (Rancans et al, 2020).

**Disclosure:**

No significant relationships.

